# Discovery of Pod Shatter-Resistant Associated SNPs by Deep Sequencing of a Representative Library Followed by Bulk Segregant Analysis in Rapeseed

**DOI:** 10.1371/journal.pone.0034253

**Published:** 2012-04-17

**Authors:** Zhiyong Hu, Wei Hua, Shunmou Huang, Hongli Yang, Gaomiao Zhan, Xinfa Wang, Guihua Liu, Hanzhong Wang

**Affiliations:** Key Laboratory of Biology and Genetic Improvement of Oil Crops, Ministry of Agriculture, Oil Crops Research Institute of the Chinese Academy of Agricultural Sciences, Wuhan, Hubei, People's Republic of China; Pennsylvania State University, United States of America

## Abstract

**Background:**

Single nucleotide polymorphisms (SNPs) are an important class of genetic marker for target gene mapping. As of yet, there is no rapid and effective method to identify SNPs linked with agronomic traits in rapeseed and other crop species.

**Methodology/Principal Findings:**

We demonstrate a novel method for identifying SNP markers in rapeseed by deep sequencing a representative library and performing bulk segregant analysis. With this method, SNPs associated with rapeseed pod shatter-resistance were discovered. Firstly, a reduced representation of the rapeseed genome was used. Genomic fragments ranging from 450–550 bp were prepared from the susceptible bulk (ten F2 plants with the silique shattering resistance index, SSRI <0.10) and the resistance bulk (ten F2 plants with SSRI >0.90), and also Solexa sequencing-produced 90 bp reads. Approximately 50 million of these sequence reads were assembled into contigs to a depth of 20-fold coverage. Secondly, 60,396 ‘simple SNPs’ were identified, and the statistical significance was evaluated using Fisher's exact test. There were 70 associated SNPs whose –log_10_ *p* value over 16 were selected to be further analyzed. The distribution of these SNPs appeared a tight cluster, which consisted of 14 associated SNPs within a 396 kb region on chromosome A09. Our evidence indicates that this region contains a major quantitative trait locus (QTL). Finally, two associated SNPs from this region were mapped on a major QTL region.

**Conclusions/Significance:**

70 associated SNPs were discovered and a major QTL for rapeseed pod shatter-resistance was found on chromosome A09 using our novel method. The associated SNP markers were used for mapping of the QTL, and may be useful for improving pod shatter-resistance in rapeseed through marker-assisted selection and map-based cloning. This approach will accelerate the discovery of major QTLs and the cloning of functional genes for important agronomic traits in rapeseed and other crop species.

## Introduction

Single nucleotide polymorphisms (SNPs) are the most abundant variations and currently an important class of genetic marker distributed throughout the genome [1]. SNPs are imperative to gene fine mapping and association studies aimed at identifying alleles that affect agronomic traits [2].

In the past decade, SNPs have been used as genetic markers in many cultivated plants such as rice [3]–[5], maize [6]–[8] and rapeseed [9]–[10]. Genotyping of SNPs has improved, making genome-wide linkage analysis and molecular breeding more rapid and efficient [5].

Bulk segregant analysis (BSA) is efficient for selective genotyping wherein DNA samples of extreme individuals from each tail of a phenotypic distribution are pooled and the two resultant bulks need to be genotyped [11]. Markers linked to a QTL affecting a particular trait are expected to be present at different frequencies in the contrasting tails, resulting in polymorphic expression of genotype signals between the two bulks [12]. BSA saves cost by only genotyping the pooled DNA from individuals with similar phenotypes. It is an effective technique in that it detects large effect QTL alleles in a large sample of progenies at a relatively low cost. Because it saves time and cost, it has been widely used for the genetic analysis of qualitative traits [12]–[14].

Reduced representation libraries (RRLs) and pyrosequencing technologies have facilitated the high throughput discovery of SNPs [15]–[19]. RRLs can be used to identify SNPs that are useful in mapping the genome for haplotypes associated with disease [15]. Genome complexity is reduced by using restriction digests and size selection to build an RRL [20]. The use of fragments from a size-selected digestion permits a similar subset of fragments to be obtained from different genotypes that can be deep-sequenced for accurate SNP discovery.

Rapeseed (*Brassica napus*; genome AACC, 2n = 38) is an allopolyploid species that originated in a limited geographic region via spontaneous hybridizations between turnip rape (*B. rapa*; AA, 2n = 20) and cabbage (*B. oleracea*; CC, 2n = 18) genotypes [21]. Rapeseed is the most important oilseed crop grown in temperate agricultural regions and has the second highest output of all oil crops in the world, providing approximately 13% of the world's supply of vegetable oil [22], [23]. Rapeseed, as a commercial oil crop in Asia, Europe, North America and Australia, has attracted interest due to its utilization in food and feed production and its growing economic importance as a novel source of renewable energy, mainly as biodiesel [24]. Rapeseed, however, is subject to significant losses in seed production as a result of premature dehiscence before and during harvest [25]. Seed shedding from pods is commonly referred to as pod shatter, which is a major production risk in rapeseed worldwide [26], [27]. Mapping quantitative trait loci (QTLs) linked to pod shatter-resistant traits will assist in developing rapeseed cultivars suitable for harvesting machinery through a molecular marker-assisted selection (MAS) strategy. Developing a large set of SNP markers for genome analyses in rapeseed will facilitate fine mapping of QTL regions and will improve the identification and exploitation of genes affecting important traits. Additionally, these makers will also allow for selective breeding through genomic selection.

In this study, our objective was to use the RRL sequencing method, via BSA, to identify SNP markers linked to rapeseed pod shatter-resistance and utilize these markers to map the QTL regions. Our study identified 70 associated SNPs, which were selected from 60,396 ‘simple SNPs’, and a major QTL was found. The QTL may be a useful tool for promoting pod shatter-resistant rapeseed through MAS and map-based cloning. Our approach will accelerate the discovery of major QTLs and the cloning of functional genes for important agronomic traits in rapeseed and many other crop species.

## Results

### Construction of RRLs

The silique shattering resistance index (SSRI) of two rapeseed lines (*B. napus* cv. zy72360 and *B. napus* cv. R1) and their F2 population progenies (including 276 individuals) were identified with a random impact test designed for screening pod shatter-resistance (Table S1). The SSRI values of zy72360 (resistance to pod shattering) and R1 (very susceptible germplasm to pod shattering) were 0.94 and 0.06, respectively. The SSRI of their F2 population progenies was continuous distribution ranging from 0.00 to 1.00, but didn't display a normal distribution. The frequency distribution of SSRI of the F2 population including 225 randomly selected individuals showed three peaks which followed 1∶2∶1 segregation ratio by chi-squared test (Fig. S1). We estimated there maybe exited one major gene controlling the pod shatter-resistance. Ten F2 individuals, which are susceptible to pod shattering (SSRI <0.10), and ten F2 individuals, which are resistance to pod shattering (SSRI >0.90), were pooled to form the susceptible bulk (SK) and the resistance bulk (RK), respectively.

**Figure 1:**
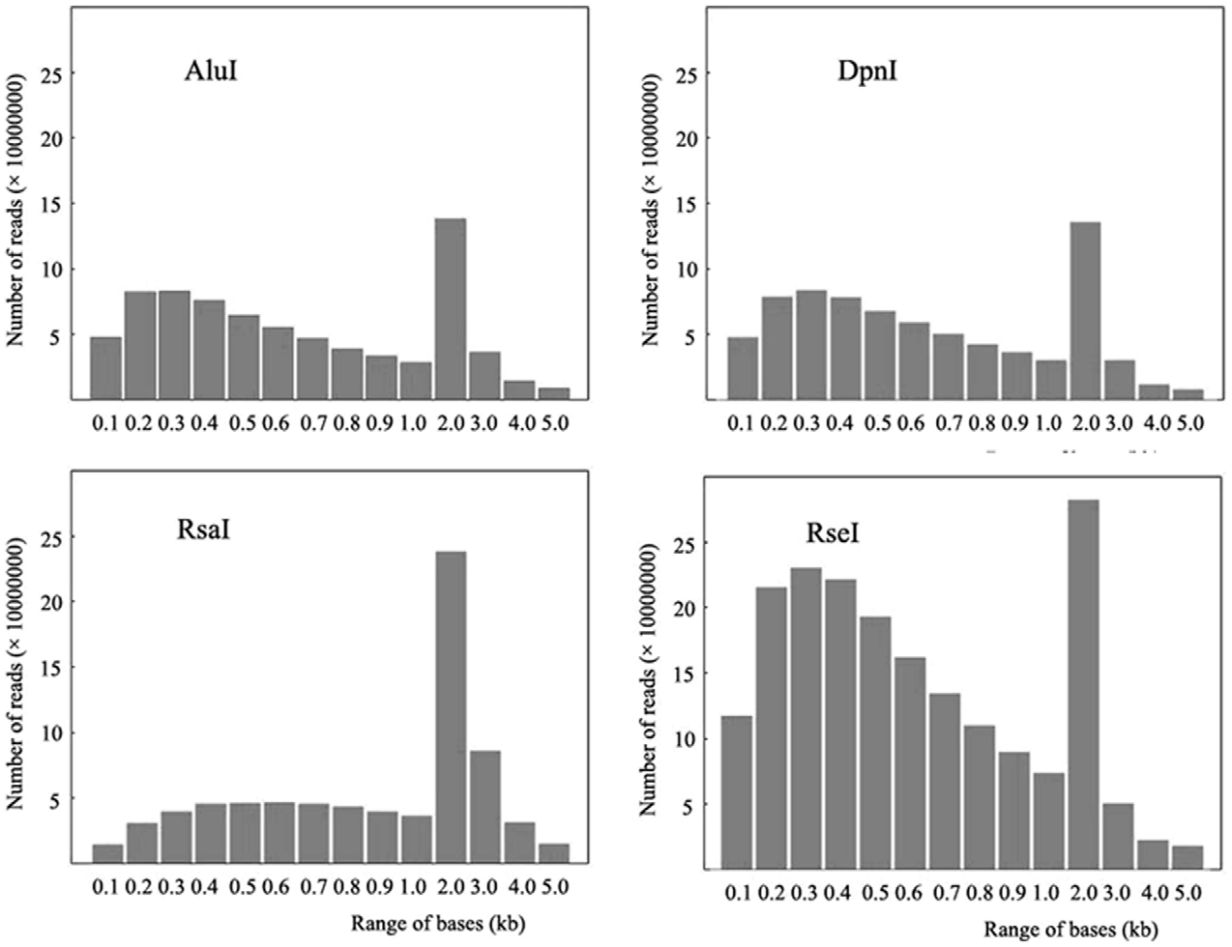
Frequency distribution of the rapeseed genome sequences. Here we show the rapeseed genome sequence after digestion with fourblunt end enzymes, *Alu*I, *Dpn*I, *Rsa*I and *Rse*I, respectively (computer simulated).

**Figure 2:**
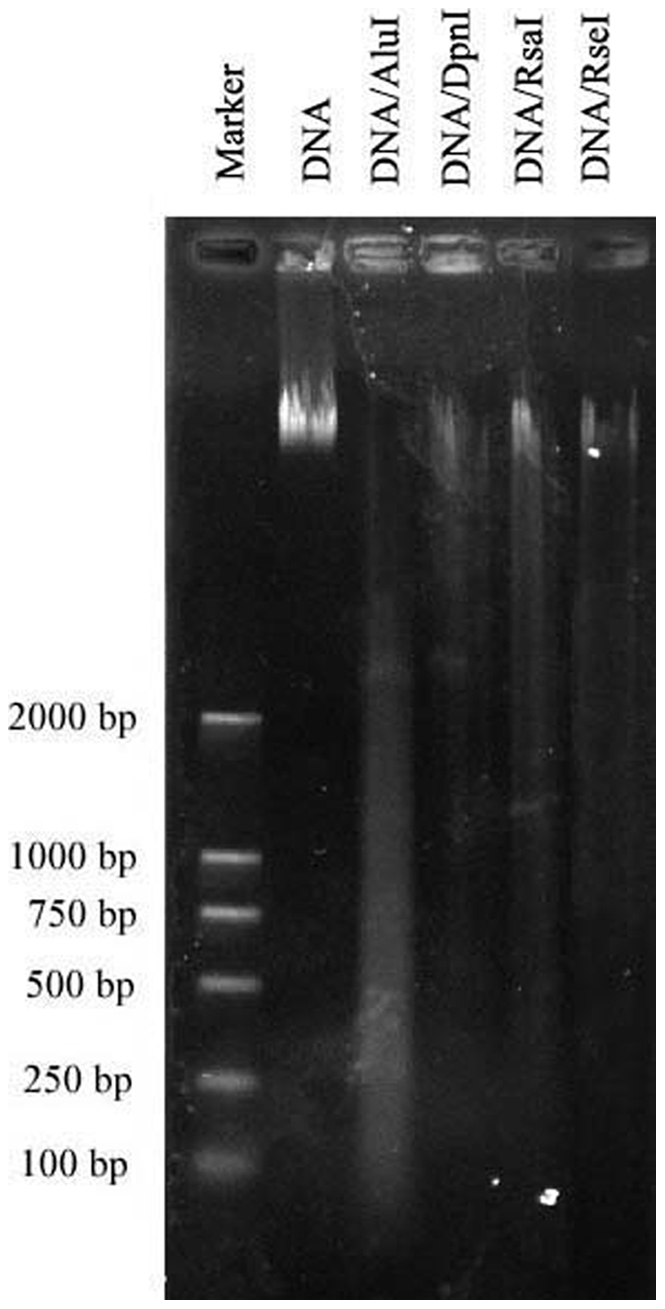
Gel image of digested rapeseed DNA. Rapeseed DNA was digested with fourblunt end enzymes, *Alu*I, *Dpn*I, *Rsa*I and *Rse*I, respectively.

To identify the most suitable enzymes for RRL construction, we analyzed all of our blunt end enzymes. Rapeseed (*B.napus*; genome AACC, 2n = 38) is a recent allopolyploid species that originated in a limited geographic region through spontaneous hybridizations between turnip rape (*B. rapa*; AA, 2n = 20) and cabbage (*B. oleracea*; CC, 2n = 18) [21]. For this reason, the combined genomes, including all sequences (790,587,732 bp) of *B. rapa* [28] and *B. oleracea* (unpublished), were used as reference genomes. Firstly, the genome sequences of *B. rapa* and *B. oleracea* were combined and the combined sequences were then simulated to be digested with all of our blunt end enzymes. In the end, four blunt end enzymes, *Alu*I, *Dpn*I, *Rsa*I and *Rse*I, were selected with the goal of minimizing repetitive content in the target size range of 450–550 bp and interpreted to be suitable for RRL construction. By digesting with these enzymes, we were assured that the size of the DNA fragments would range from 450 to 550 and account for about 10% of the size of the whole sequences (Figure 1). After validation by using megabase-size rapeseed genomic DNA, we found that the restriction enzymes *Dpn*I, *Rsa*I and *Rse*I did not entirely digest the rapeseed DNA (Figure 2) and were therefore not considered for further procedures. *Alu*I, an enzyme that completely digests rapeseed DNA (Figure 2), was ultimately selected for construction of the library with the target size range of 450–550 bp from the genome and to obtain a suitable quantity for solexa sequencing.

### Sequencing Results

We independently sequenced the two RRLs (SK and RK), and a total of 48,888,892 reads (90 bp) were obtained (Table 1). Because we prepared the libraries using *Alu*I, properly ligated fragments included a two-nucleotide CT tag at each restriction site. We discarded the 1,031,506 sequences that did not begin with this tag, leaving ∼ 48 million reads for analysis. Because rapeseed genome has not been pieced together, the combined genomes, including all sequences (790,587,732 bp) of *B. rapa* and *B. oleracea*, were used as reference genomes. Subsequently, the sequences were mapped to the reference genomes using SOAP software. We discarded reads that matched exactly with more than one position in the reference genomes, and the unique sequences were chosen for SNP discovery. The most frequently occurring sequence (798,774 occurrences) was a perfect match to more than 100 locations in the reference genomes and represented a contaminating repetitive element. After removing 1,102,886 sequences with low-quality scores, 47,786,006 reads (4.3 billion bp) corresponding to 25,048,721 (12,633,495 and 12,415,226 from RK and SK libraries, respectively) unique sequences remained for SNP discovery.

**Table 1:** Summary of DNA sequence production and filtering.

			Edits		
Library	Total sequences	CT present^a^ (%)	Pass quality control^b^ (%)	Mapped reads	Unique reads
RK	24,444,448	23,813,952 (97.4)	23,778,699 (97.3)	21,431,250 (87.7)	12,633,495 (51.7)
SK	24,444,444	24,043,434 (98.4)	24,007,307 (98.2)	21,356,158 (87.4)	12,415,226 (50.8)
Total	48,888,892	47,857,386 (97.9)	47,786,006 (97.7)	42,787,408 (87.5)	25,048,721 (51.2)

a Sequences remaining after filtering those without a CT tag at the restriction site. ^b^Sequences remaining after sequentially filtering those that failed quality control (see the Methods section).

The sequences obtained from SK and RK libraries covered 14% (114,308,963/790,587,732) and 11% (84,456,839/790,587,732) of the reference genomes, respectively. The overlap of the two libraries covered 8% (66,622,234/790,587,732) of the reference genomes and achieved a 20-fold average coverage depth, and that coverage depth was sufficient for SNP identification.

### Associated SNP Discovery

The unique sequences produced ‘simple SNPs’ between two bulks by custom perl scripts [9] and 60,396 ‘simple SNPs’ were obtained in total. The statistical significance level was evaluated using Fisher's exact test [29]. Our results indicated that there were 25,226, 3,573, 450 and 70 ‘simple SNPs’remained when the value of –log_10_ *p*was over 2, 5, 10 and 16, respectively. In order to reduce the number of associated SNPs in our study, only those putative associated SNPs with the value of –log_10_ *p*over 16 were selected for further analysis. Thus, these 70 SNPs associated with rapeseed pod shatter-resistance were obtained and listed in Table S2.

### Mapping and Distributionof Associated SNPs

Using the genome sequences from *B. rapa* and *B. oleracea*, 70 associated SNPs were mapped to the reference genomes using SOAP software (Table S2) [30]. The map of the SNP distribution within the rapeseed genome was drawn using Microsoft Excel software (Figure S2). 70 associated SNPs were distributed on 17 rapeseed chromosomes. The map reveals the appearance of a tightly clustered SNP region which included 37% (26 SNPs) of the associated SNPs on chromosome A09 (Figure S2, Table S2). Furthermore, the location of 20% (14 SNPs) associated SNPs were located within a 396 kb sequence on chromosome A09 (Figure S2, Table S2). All the other SNPs did not appear in an obvious cluster. Because these SNPs were discovered using the two libraries, RK and SK, with large phenotype differences in rapeseed pod shatter-resistant traits, the tightly clustered region of associated SNPs implies that there likely exists a QTL for rapeseed pod shatter-resistance on chromosome A09.

### QTL Location and Associated SNP Validation

**Figure 3:**
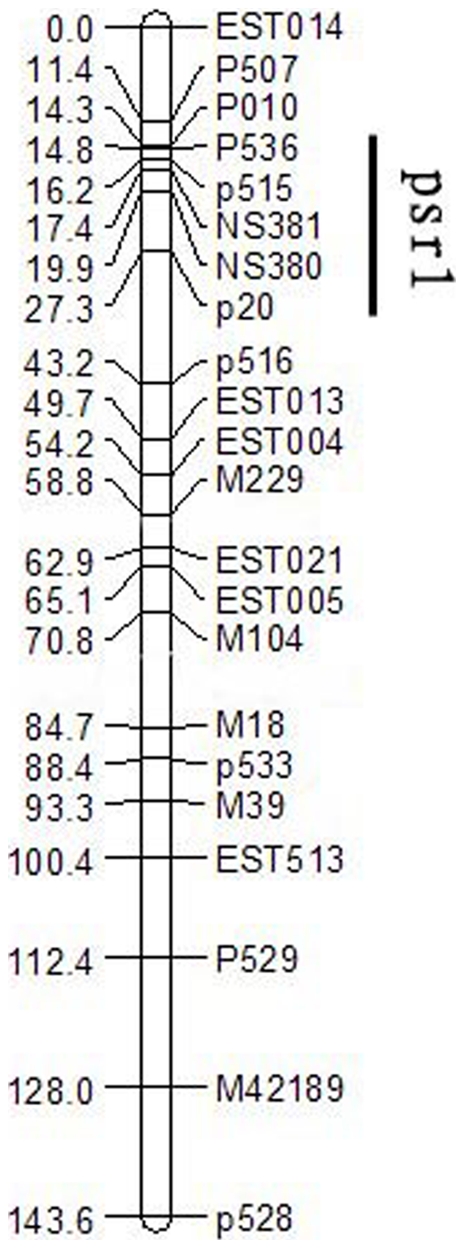
Genetic linkage map of chromosome A09 from rapeseed. The numerals in left column indicate the genetic distance [centiMorgan (cM)]. SNP or SSR marker names are indicated in the right column. *Psr1* indicates the QTL region of pod shatter-resistance.

In this study, we simultaneously constructed a genetic map by using the F2 population derived from the cross of zy72360 and R1. The SSRI of 276 individual pods from the F2 population was measured by random impact test according to the previously described method [31] with minor modifications. The QTL was scanned by WinQTLCart software [32] and one QTL, *psr1*, which influenced pod shatter-resistance was detected (Fig. 3, Fig. S3). The QTL allele, *psr1*, is located just on chromosome A09 and its genetic contribution rate is 47%. This result is consistent with that from associated SNPs and indicates that the associated SNP clustered region was indeed convenient for marking the location of the QTL.

In order to further validate if the location of the tightly clustered SNP region was identical to the QTL allele, *psr1*, 14 primer pairs were designed and synthesized from the 14 SNPs within the tightly clustered SNP region on chromosome A09 (Table S3). After polymorphic SNP primers were designed from the SNP flanking sequences of the reference genomes, amplicons were generated from the F2 population (derived from the cross of zy72360 and R1), genotyped using non-denaturing polyacrylamide gels and validated for sequence variations. Surprisingly, two associated SNP markers, NS380 and NS381 were mapped and traced to the region of the QTL allele, *psr1* (Fig. 3).

## Discussion

In this study we chose rapeseed pod shatter-resistance as the target trait, and succeeded in isolating the associated SNP markers and defining a major QTL region by using a method that combines BSA, RRL and sequencing. Our results are proof of its easy applicability and of its validity, indicating that it is a rapid and low-cost method for the discovery of associated SNP markers. BSA was first developed by Michelmore et al. [11] as a method for rapidly identifying markers linked to any specific gene or genomic region. Feng et al. [33] identified three markers linked to the *or* gene in *B. rapa* by BSA. Xu et al. [34] and Huang et al. [35] employed AFLP technology combined with BSA to identify markers linked to the seed color gene in *B. juncea* and the *BnMs3* gene in *B. napus*, respectively. By applying high-throughput sequencing to BSA, Wenger et al. [36] revealed a novel xylose utilization gene from *Saccharomyces cerevisiae*. BSA has also been used for the fine mapping and for shortening the confidence intervals of QTL regions [13], [37]. RRL coupled with next generation sequencing technology allows for the detection of a large number of SNPs at a much lower time investment and expense than by conventional methods, and is used widely in animal and plant species such as cattle [18], swine [19], rainbow trout [2] and soybean [20].

The key technological aspects of our study include the selection of a versatile enzyme for RRL construction and the preferences for associated SNP selection. The selection of an optimal blunt end enzyme is the most important for RRL construction. In animal studies, the restriction enzyme *Hae*III was usually selected for RRL construction in cattle [18], swine [19] and rainbow trout [2]. However, in plant studies, Hyten et al [20] reported that four different blunt end restriction enzyme combinations, each containing five different restriction enzymes, were tested as an effort to reduce the likelihood of repetitive sequences. The authors created a soybean RRL that consisted of DNA digested with a combination of *Hae*III. *Psi*I, *Ssp*I, *Rsa*I and *Msl*I. We simulated the digestion of the masked rapeseed sequences on a computer using all of our blunt end enzymes. From this analysis, we decided to use *Alu*I due to its excellent *in silico* digestion characteristics. Our results indicate that *Alu*I is optimal for the construction of *B. napus* RRLs, and this was evident because almost 98% of our sequence reads began with the ‘CT’ sequence (Table 1).

In this study, the use of RRL, coupled with next generation sequencing and BSA, provided a powerful method for the discovery of SNP markers linked to agronomic traits.The advantage of our method is that it is rapid and requires a low cost to obtain associated SNPs and find major QTLs. Schneeberger et al. also report a similar method, SHOREmap, in which they identify a causative mutation in only 8 working days of hands-on time, and can also be applied to mapping of a QTL. Their method, however, is very expensive as it used a single genomic DNA sample to prepare an Illumina library, which was sequenced to 22-fold genome coverage [38]. In our method, the sequences used were only about 10% of the size of the whole sequences of the RRLs, and the costs were greatly reduced. In this study, we only analyzed sequences of about 500bp, the target size range for RRL regions. The larger the target size of the RRL, the less sequences were needed for adequate coverage. If the target size range for the RRL was increased from 500 bp to 2 kb, then only about 25% of the sequencing power was needed to reach the same average coverage depth, and the costs were reduced by at least a half. For later studies in which we look at other species or other traits, we will consider different target size combinations depending on the enzyme that is used for RRL construction and on the genome size of the particular species in question. One major disadvantage of our method is that it cannot be precisely used to evaluate the genetic contribution of each QTL region. In our study, we only identified one major QTL for pod shatter-resistance and therefore we only discovered one tightly clustered SNP region. In theory, for traits containing several QTLs, the degree of QTL contribution is suggested by the frequency of the associated markers.

Fully mature rapeseed pods are extremely sensitive to opening, resulting in seed loss. This loss is typically 8–12% of the total seed yield [39], and can even exceed 20% if harvesting is delayed beyond the optimum time or under adverse conditions [26]. Thus, rapeseed pod shatter-resistance is a very important agronomic trait. In this study, a total of 70 SNPs associated with rapeseed pod shatter-resistance were identified. The most tightly clustered SNP region, which contains 14 associated SNPs within a 396 kb stretch on chromosome A09, indicates and validates the existence of the large effect QTL allele, *psr1*. The SNP markers closely linked to pod shatter-resistance were applied to fine mapping of the QTL and are useful for pod shatter-resistant improvement in rapeseed through MAS and map-based cloning. Based on its quick application and low cost, our approach can be extensively applied in discovering major QTLs and will accelerate the cloning of functional genes for important agronomic traits in rapeseed and other crop species.

## Materials and Methods

### Ethics Statement

No specific permits were required for the described field studies. No specific permissions were required for these locations/activities. The location is not privately-owned or protected in any way. The field studies did not involve endangered or protected species.

### Plant Materials

Two rapeseed lines, *B. napus* cv. zy72360 (resistance to pod shattering) and *B. napus* cv. R1 (very susceptible germplasm to pod shattering) were selected in this study. An F2 population that included 276 individuals derived from the cross of zy72360 and R1 was used. All plant materials were planted in a field located in Wuhan, China. All lines were planted in fields and each condition duplicated.

### Random Impact Test

Random impact test was conducted according to the method in [31] with minor modifications. Ten replicates of 20 intact mature pods were placed in a closed polythene drum of 10 cm diameter, 14 cm height with fifty steel balls of 8 mm diameter. The drum was then shaken mechanically in a rocking bed (HQ45Z, Wuhan Scientific Instrument Factory, China) at 300 rpm for 1 min. The drum was opened after each replicate and the number of broken and damaged pods was counted. The SSRI was counted according to the following expressions:
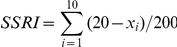
Where x_i_ is the number of broken and damaged silique at each replicate, i. Each accession was tested twice and an average value was calculated.

### DNA Isolation and Pooling of Samples for BSA

Genomic DNA was isolated from fresh leaves using cetyl trimethyl ammonium bromide. The concentration of DNA was adjusted to 50 ng/µL for PCR amplification. Megabase-size genomic DNA was isolated from bulk leaves of F2 individuals following the procedure described by Zhang et al. [39] with minor modifications. Ten F2 plants, which are susceptible to pod shattering (SSRI <0.10), and ten F2 plants, which are resistance to pod shattering (SSRI >0.90), were pooled to form the SK and RK bulks, respectively.

### RRL Construction and Sequencing

Four different restriction enzymes (*Alu*I, *Dpn*I, *Rsa*I and *Rse*I), producing fragments with blunt ends, were tested for library construction. Digestion of DNA was performed for each enzyme as suggested by the manufacturer (New England Bio Labs) and was digested overnight to ensure complete digestion. The digested DNA was then run on a 2% agarose gel and the digestion products were excised from the agarose gel at the 450 to 550 bp region. The QIAquick Gel Extraction Kit (Qiagen, Hilden, Germany) was used following the manufacturer's protocol to obtain a total of 5,000 ng of size-selected DNA. The blunt-end DNA fragments were given to BGI (Beijin Genome Institute, Shenzhen, China) for sequencing on the HiSeq2000 (Illumina, Inc; San Diego, CA, USA) after adding a 10 bp index. The sequence data obtained from BGI contained 90 bp sequence tags in which every base was given a quality score that is similar to a Phred score. We screened the data for overall quality by looking for an average base quality score of at least 25 across the entire tag for all reads according to a previously described method [18]. The Illumina sequence data have been deposited in the NCBI, Sequence Read Archive [GenBank: SRA045576].

### Associated SNP Marker Discovery

Rapeseed is a recent allopolyploid species that originated in a limited geographic region through spontaneous hybridizations between *B. rapa* and *B. oleracea* genotypes [21]. In this study, a total of 790,587,732 bp sequences which include the combined genomic sequence of *B. rapa* [28] and *B. oleracea* (unpublished) were used as reference genomes. The sequences from the two bulks were mapped to the references using SOAP software [30]. The results produced ‘simple SNPs’ between the two bulks by custom perl scripts [9]. The statistical significance level was evaluated using Fisher's exact test [29].

### Linkage Analysis

The F2 population, consisting of 276 individuals, was utilized to construct a genetic map and validate the associated SNPs. The primers sequences which were used for the genetic map are listed in Table S4. PCR amplification was carried out in a solution of 20 μL containing 100 ng of genomic DNA, 6 pmol of each primer, 2.5 mmol/L of MgCl_2_, 0.25 mmol/L of each dNTP, ×reaction buffer and 0.75 U Taq polymerase. Thermocycling was performed using a DNA Engine Peltier Thermal Cycler, with 30 cycles of 94°C for 30 s, 55°C for 30 s, 72°C for 1 min, and a final extension at 72°C for 5 min before holding at 10°C. The PCR products were separated on an 8% non-denaturing polyacrylamide gel, and were stained with AgNO_3_ [40].

Linkage analysis and genetic map construction was done using JoinMap 3.0 [41]. The QTL was scanned using WinQTLCart software [32].

## Supporting Information

Figure S1 **The frequency distribution of the silique shattering resistance index.** The three dashed frames show three peaks.
(TIF)

Figure S2 **Distribution of associated SNPs detected among rapeseed SK and RK bulks.** The star indicates the tightly clustered SNPs region.
(TIF)

Figure S3 **Plot of the major QTL for pod shatter-resistance on linkage group A9 in** ***B. napus*** **.**
(TIF)

Table S1 **The silique shattering resistance index of two rapeseed lines (** ***B. napus*** **cv. zy72360 and** ***B. napus*** **cv. R1) and their F2 population progenies (including 276 individuals).**
(XLS)

Table S2 **The details of 70 SNPs associated with rapeseed pod shatter resistance from** ***B. napus*** **.**
(XLSX)

Table S3 **Primers of 14 SNPs of the tightly clustered SNPs region on A09 chromosome.**
(XLSX)

Table S4 **Primers sequences of the genetic map on A09 chromosome.**
(XLSX)
