# Supplementary figures and images for: Discovery of Pod Shatter-Resistant Associated SNPs by Deep Sequencing of a Representative Library Followed by Bulk Segregant Analysis in Rapeseed
Source: PLoS One. 2012 Apr 17;7(4):e34253. doi: 10.1371/journal.pone.0034253 (PMC3328475; doi:10.1371/journal.pone.0034253)

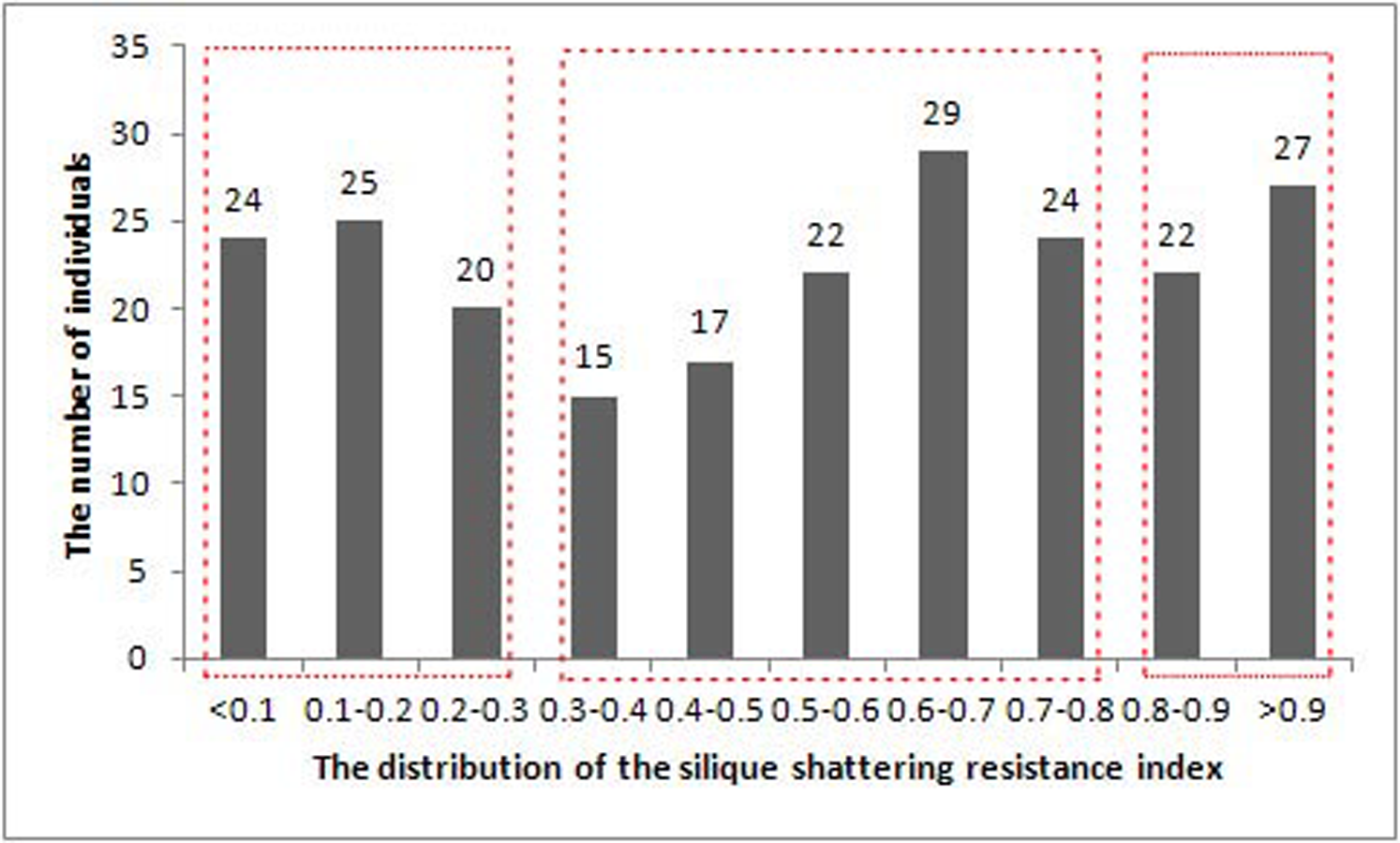

Supplement: Figure S1 — The frequency distribution of the silique shattering resistance index. The three dashed frames show three peaks. (TIF) [file pone.0034253.s001.tif]

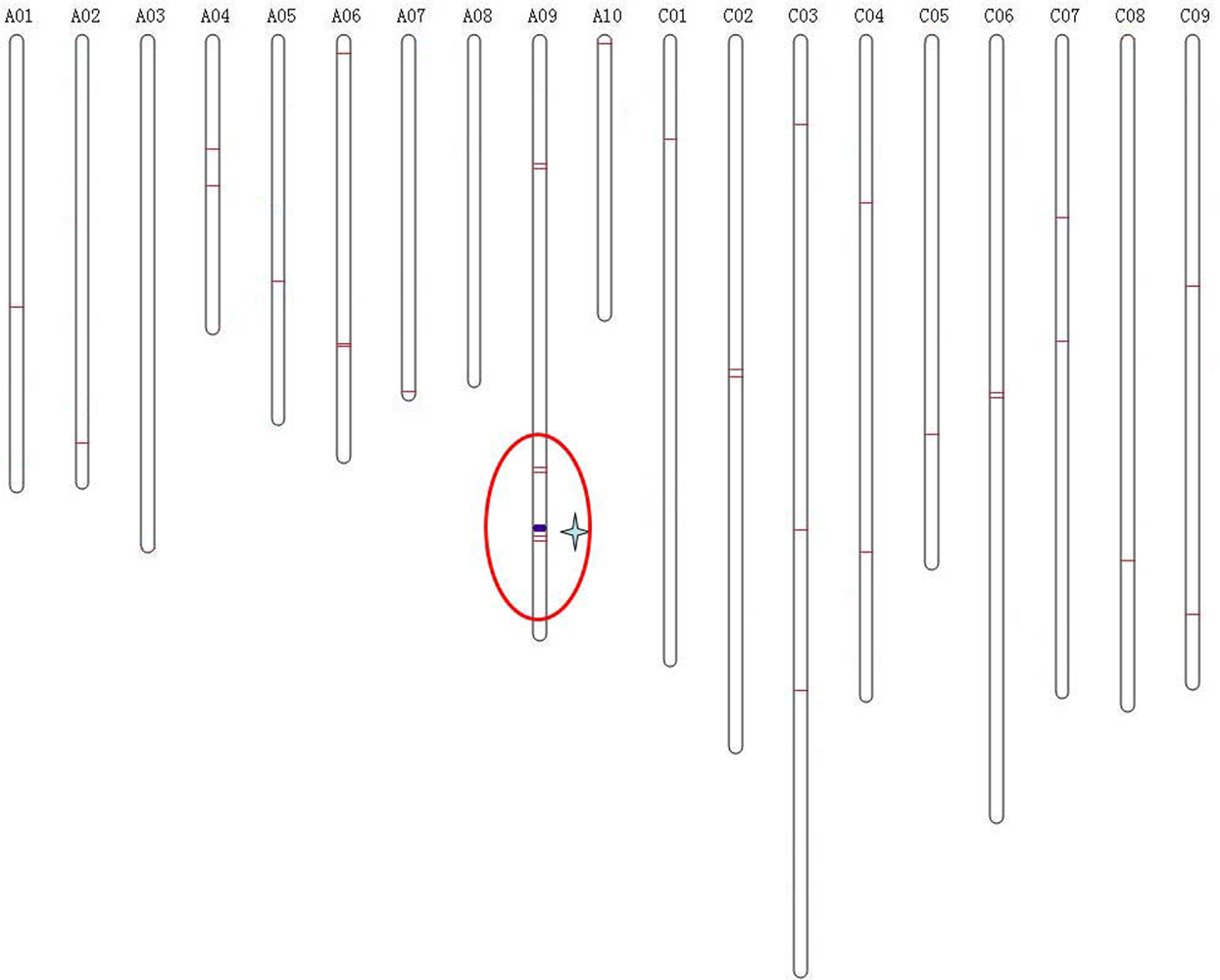

Supplement: Figure S2 — Distribution of associated SNPs detected among rapeseed SK and RK bulks. The star indicates the tightly clustered SNPs region. (TIF) [file pone.0034253.s002.tif]

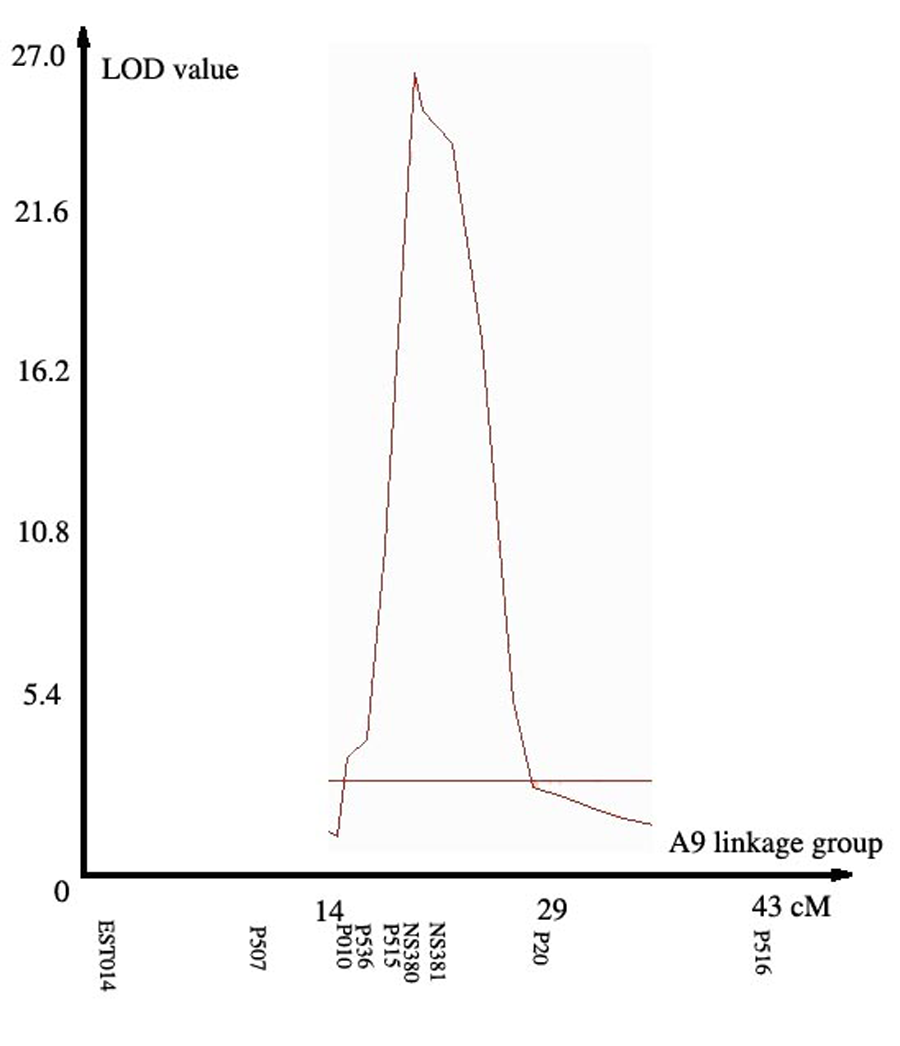

Supplement: Figure S3 — Plot of the major QTL for pod shatter-resistance on linkage group A9 in B. napus . (TIF) [file pone.0034253.s003.tif]
